# Supplementary material for: Extraction of higher-order nonlinear electronic response in solids using high harmonic generation
Source: Nat Commun. 2019 Jul 22;10:3272. doi: 10.1038/s41467-019-11096-x (PMC6646338; doi:10.1038/s41467-019-11096-x)
Supplement: Supplementary file 1 — Supplementary info [file 41467_2019_11096_MOESM1_ESM.pdf]

Supplementary Information

Extraction of higher-order nonlinear electronic response in solids using high harmonic generation

Han et al.

Supplementary Note 1:

In the following we describe the calculation of the absolute values of the nonlinear susceptibilities, which requires determination of physical units of the HHG yield from the experiments as well as all the constants contributing to the prefactor A in $Yield = A \cdot I^N$.

Conversion of average power into peak intensity

The peak intensity (I_{peak}) of the incident laser pulse is determined by the formula below;

$$I_{\text{peak}} = \frac{P}{f_{\text{rep}} \times \tau_{\text{p}} \times A}$$

P is the average power of the incident laser pulses and is tunable from 0 to 370 mW in this experiment. f_{rep} is the pulse repetition rate, τ_{p} is the pulse duration, and A is the focal area. The average power of incident laser pulses at 370 mW corresponds to a peak intensity of 13 TW·cm^{-2} in vacuum. The peak intensity in material is obtained through the following equation considering reflectance at the interface,

$$I_{\text{material}} = \frac{4n}{(1+n)^2} I_{\text{vacuum}}$$

where n is the refractive index of sapphire (1.76). As a consequence, the intensity in sapphire is reduced to ~12 TW·cm^{-2} when maximum average power was set for the driving laser. Converting to field strength, this results in a value of 0.72 V·Å^{-1} within the sapphire substrate.

HHG power calibration

The unit of the measured spectral intensity from the micro-channel plates in the spectrometer is arbitrary. We can convert the arbitrary spectral intensity to the photon rate in unit of generated photon number per second by measuring the photocurrent induced by the harmonics. The photomultiplier tube responds to EUV radiation with wavelength range of 30-150 nm, which corresponds to harmonic orders from 7 to 25. A Cu-BeO photomultiplier tube (R595, Hamamatsu) was placed 20 mm from the samples, and the photocurrent (I_{PMT}) was measured using a picoammeter (6485, Keithley). The photon rate (N) was estimated using the following formula.

$$N = \frac{I_{\text{PMT}}}{q \times Q \times G \times \eta}$$

q indicates the electron charge (1.6 x 10^{-19} C), Q is the quantum efficiency of the photomultiplier tube for measured spectral range, G is the amplification gain of the photomultiplier tube (4×10^5), and η is the collection efficiency that can be assumed unity due to the low divergence of the generated harmonics. The effective Q is calculated by considering the spectral quantum response of the photomultiplier tube and the photon flux ratio of harmonics from the measured spectral intensity by the spectrometer. We assumed that the measured harmonics from the photomultiplier tube only consist of the order of 7, 9, and 11 because the number of photons of harmonic orders above 13 are negligible. The photon rates of these harmonics are measured to be larger than 10^7 photons per second for an incident peak intensity of 1.78 TW·cm^{-2}.

The photon rate of harmonics can be transformed into the power from the following equation.

$$E = \frac{N \times h \times c}{\lambda}$$

h denotes the Planck constant, c is the speed of light, and λ the wavelength. The power of harmonics is estimated to be more than several tens of pico-watt level.

Determination of effective volume of high harmonics generation

We assumed that the focal volume has the shape of a cylinder with a height of 20 nm and a diameter of 2 μm respectively since the EUV produced by high harmonic generation is reabsorbed by the sapphire itself. The spot size is estimated with a consideration of lens parameters, laser beam size, and cut-off of high harmonic spectrum provided by theory. The transmittance of light through sapphire with thickness of 20 nm is estimated to be 0.10 for the 7th, 0.22 for the 9th, and 0.36 for the 11th harmonic. Therefore, the harmonics generated from depths above 20 nm from the sapphire surface are re-absorbed significantly before escaping the samples, resulting in no substantial contribution to the harmonic yield.

Spectral modulation at 7th order harmonic generation

We experimentally observed spectral modulation of high harmonic generation at 7th harmonics for the different pump laser intensities. This effect was weakly observed at low pump laser intensity and start to appear at high pump laser intensity which influences the fitting HHG yields to I^7 fitting line respectively.

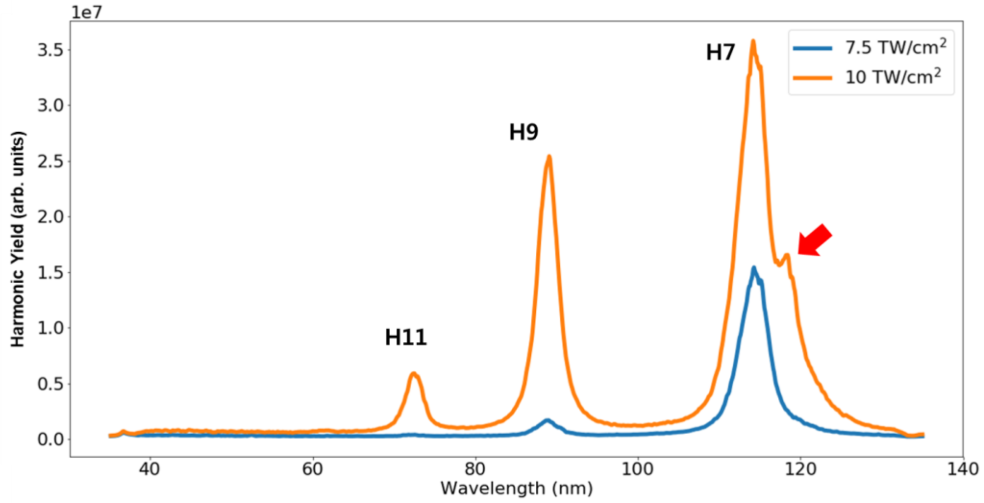

Supplementary Figure 1. Experimental data: High harmonic spectrum at different laser intensities. The red arrow indicates the spectral modulation (or splitting) of harmonics that is experimentally observed at 7th order harmonic.

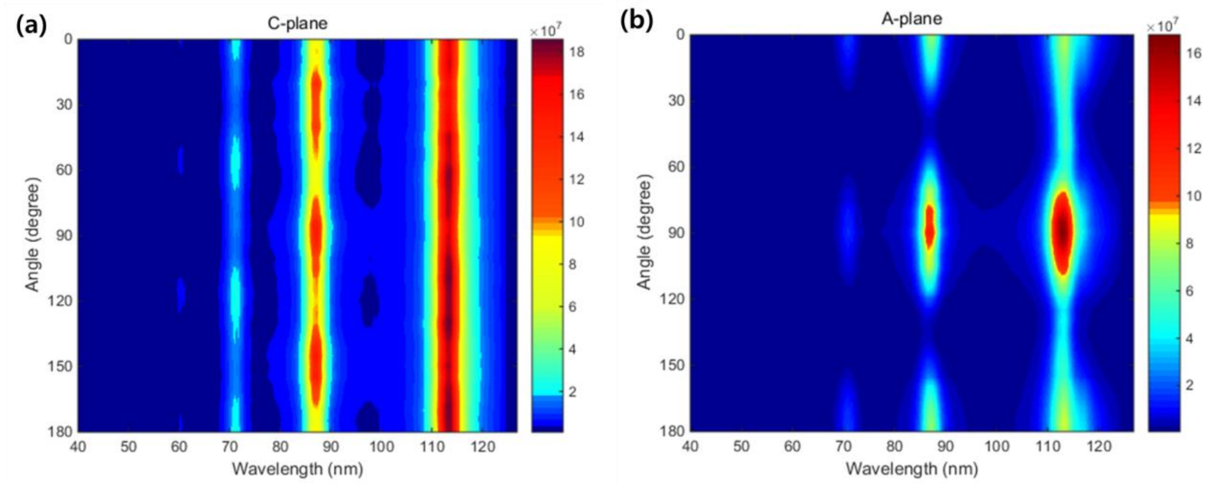

Supplementary Figure 2. Periodicity of generated high harmonics generation for two different planes. High harmonic spectrum at different crystal orientations. a) for c-plane sapphire b) for a-plane sapphire. The same intensity scale bar was used for plotting all high harmonics intensities.

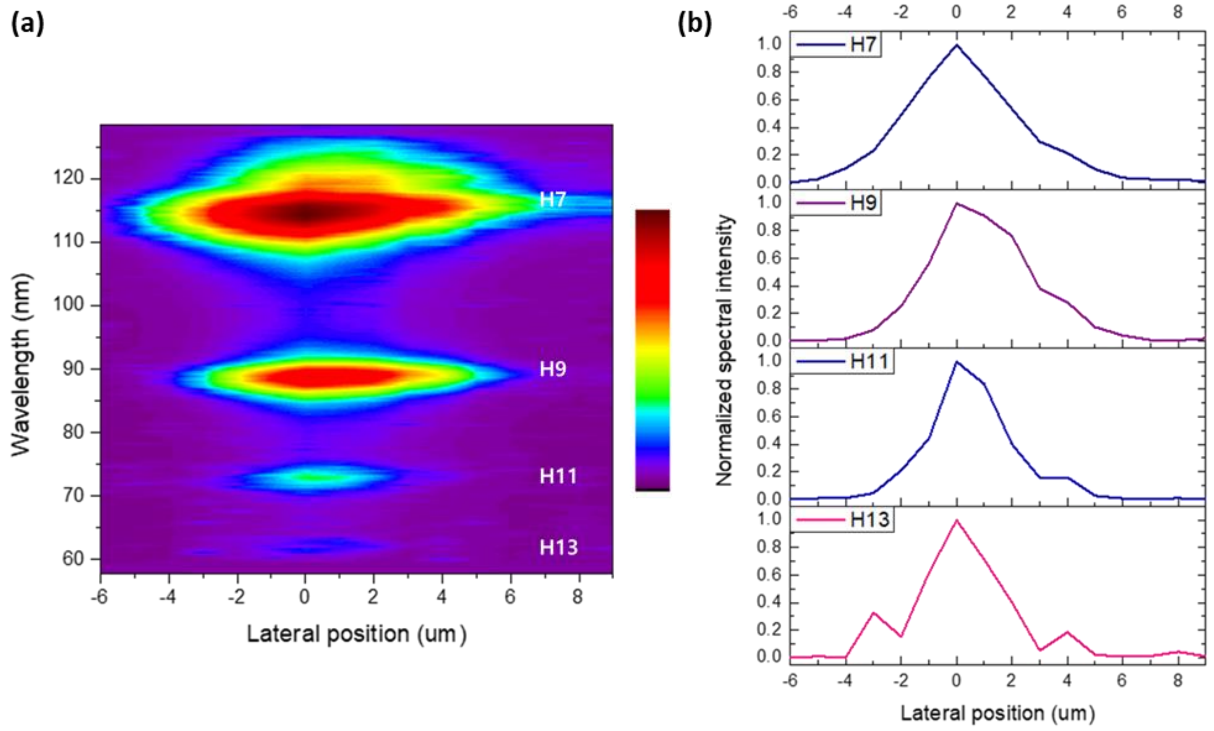

Supplementary Figure 3. Measured power of the generated high harmonics at different focal position. a) for all spectral components b) extracted plot of center wavelength of each harmonic. The lateral position of 0 is near the surface of the sapphire and the negative value implies that focal position is within the sapphire substrate whereas the positive value means the focal position at air.

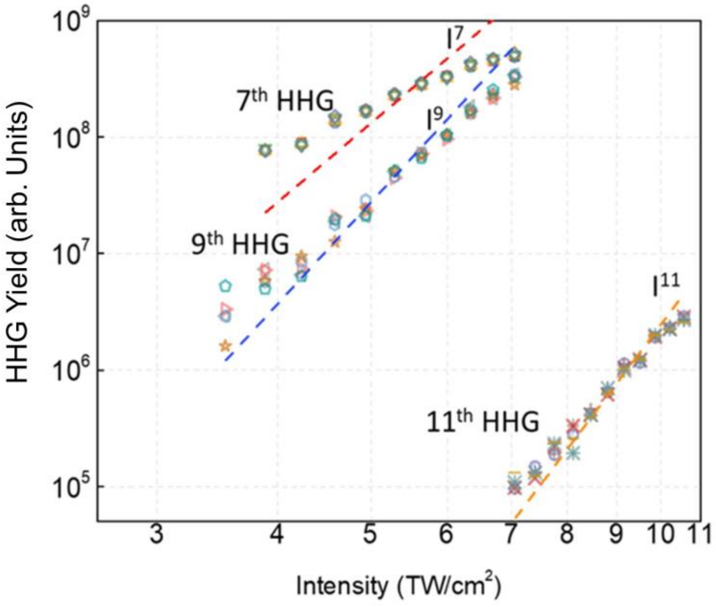

Supplementary Figure 4. The experimental data on a log-log scale: The experimental yield as a function of intensity on a log-log scale for the three harmonic orders under consideration along Γ-K direction. Each harmonic yield is measured five times at the same pump laser intensities for fitting the harmonic yields.

C-plane	Γ-K	Γ-M	Γ-K	Γ-M	Γ-K	Γ-M	Γ-K
Harmonic order	0˚	30˚	60˚	90˚	120˚	150˚	180˚
7	0.931	0.873	0.980	0.933	0.981	0.965	0.986
9	0.584	0.758	0.547	0.969	0.625	0.941	0.606
11	0.907	0.513	0.893	0.636	0.973	0.649	0.912
13	0.894	0.169	0.890	0.237	0.932	0.276	0.907

A-plane	Γ-A	Γ-M	Γ-A
Harmonic order	0˚	90˚	180˚
7	0.466	1.000	0.5
9	0.593	1.000	0.576
11	0.978	0.876	1.000
13	0.764	1.000	0.833

Supplementary Table 1. Normalized high harmonic yield for different crystal orientations: The high harmonic yield is normalized by the maximum value per each harmonic order for all crystal orientations. For C-plane sapphire, H7, H11, and H13 has maximum harmonic yields at Γ-K direction, but maximum H9 yield is observed at Γ-M direction. All harmonic yields for C-plane sapphire show six-fold symmetry. For A-plane sapphire, H7, H9, and H13 has maximum harmonic yields at Γ-M direction and H11 at Γ-A direction. All harmonic yields for A-plane sapphire show two-fold symmetry.

Supplementary Note 2.

The results shown in Fig. 3 (a), (c) and (d) from the main text follow the model presented in [1], which is based on Bloch equations. In our case the band structure of sapphire for the three different crystal orientations (laser pointing in Γ-A, Γ-K, or Γ-M direction) was modeled using DFT calculations under the assumption of a 1D band (using the direction parallel to the laser polarization direction), and the band gap energy was taken from the previously measured references [2]. For the calculations in Fig. 3 (a) and (c) in the main text, the valence bands were fitted by a single cosine

$$E_i(k) = I_{v0,i} - I_{v,i} cos(ka_i) \quad (1)$$

with the following coefficients:

	$I_{v0,i}$	$I_{v,i}$
Γ-K, valence band 3	-0.03872851	-0.01890649
Γ-K, valence band 5	-0.04973077	-0.01625438
Γ-M, valence band 3	-0.0273576	-0.01318281
Γ-K, valence band 5	-0.03746373	-0.01007603
Γ-A, valence band 1	-0.01715089	-0.00262927

Supplementary Table 2. Fitting parameters for different valence bands: Fitting parameters obtained from the fit of supplementary equation (1) to the valence bands in three crystal directions.

The conduction bands were fitted by the sum

$$E_i(k) = \sum_{n=0}^{6} \epsilon_{n,i} cos(nka_i) \quad (2)$$

with fitting constants $\epsilon_{n,i}$ and lattice constants a_i in the directions i={Γ-A, Γ-K, Γ-M}. The cosine sum takes account of intraband HHG emission depending crucially on the electrons oscillating in the conduction band producing nonlinear currents. The parameters obtained from the fit for the first conduction band, the transition to which dominates, are given in supplementary Table 3 and the lattice constants are $a_{\Gamma-M} = 4.11$ Å, $a_{\Gamma-K} = 3.56$ Å and $a_{\Gamma-A} = 12.96$ Å.

	i=Γ-M	i=Γ-K	i=Γ-A
$\epsilon_{0,i}$	0.2360	0.2428	0.1945
$\epsilon_{1,i}$	-0.0514	-0.0550	-0.0074
$\epsilon_{2,i}$	0.0061	0.0020	0.0017
$\epsilon_{3,i}$	-0.0036	-0.0020	-0.0007
$\epsilon_{4,i}$	0.0019	0.0009	0.0004
$\epsilon_{5,i}$	-0.0012	-0.0006	-0.0003
$\epsilon_{6,i}$	0.0009	0.0004	0.0002

	i=Γ-M	i=Γ-K	i=Γ-A
$\epsilon_{2,i}/\epsilon_{1,i}$	0.119	0.063	0.225
$\epsilon_{3,i}/\epsilon_{1,i}$	0.070	0.065	0.101
$\epsilon_{4,i}/\epsilon_{1,i}$	0.037	0.028	0.057
$\epsilon_{5,i}/\epsilon_{1,i}$	0.024	0.019	0.037
$\epsilon_{6,i}/\epsilon_{1,i}$	0.017	0.012	0.026

Supplementary Table 3. Fitting parameters for the first conduction band: Fitting parameters obtained from the fit of supplementary equation (2) to the first conduction bands in three crystal directions (left panel). We find that the ratio of higher order fitting parameters ($i \geq 2$) and the first order fitting parameter ($i = 1$) (right panel), is larger for the Γ-A direction than it is the case for the Γ-M and Γ-K direction, which indicates the higher anharmonicity in the Γ-A direction.

For Γ-K and Γ-M, the transition dipoles to the second conduction band are not negligible and therefore, these conduction bands were included in the calculation as well. The fitting coefficients for these bands are

	i=Γ-M	i=Γ-K
$\epsilon_{0,i}$	0.2737	0.2771
$\epsilon_{1,i}$	-0.0317	-0.0322
$\epsilon_{2,i}$	0.0006	-0.0027
$\epsilon_{3,i}$	0.0003	0.0005
$\epsilon_{4,i}$	-0.0007	-0.0003
$\epsilon_{5,i}$	0.0007	0.0002
$\epsilon_{6,i}$	-0.0006	-0.0001

Supplementary Table 4. Fitting parameters for the second conduction band: Fitting parameters obtained from the fit of the supplementary equation (2) to the second conduction bands in the Γ-M and Γ-K direction.

The dipoles of the transition that were taken into account for these calculations as well as for the calculations presented in Fig. 3 (b) from the main text are:

Γ-M (v=3→c=1): 0.73972

Γ-M (v=5→c=2): 0.47742

Γ-K (v=3→c=1): 0.70793

Γ-K (v=5→c=2): 0.47717

Γ-A (v=1→c=1): 0.74347,

where v denotes the valence band number, with v=1 corresponding to the highest lying valence band, and c specifies the conduction band number, with c=1 corresponding to the lowest lying conduction band.

Note that even though the transition dipoles do not seem to differ much between the transitions to the first and second conduction band, this small difference matters a lot since the transition dipoles affects the yield in the 4th power.

Following the equations and notation in [1] the calculations shown in Fig. 3 (a) and (c) from the main text were done shifting eq. (1) and (2) in [1] to the moving reference frame ($\acute{BZ} = BZ - A(t)$), which allows us to remove the term including ∇_k. We can thus write the semiconductor Bloch equations as

$$\acute{\pi}(K,t) = -i\left[\epsilon_g\big(K + A(t)\big) - \frac{i}{T_2}\right]\pi(K,t) - iE(t)\cdot d\big(K + A(t)\big) \tag{3}$$

$$\acute{n}_m(K,t) = is_m E(t)\cdot d\ \ \big(K + A(t)\big)\pi(K,t) - is_m E(t)\cdot d\big(K + A(t)\big)\pi\ \ (K,t) \tag{4}$$

where we set $w(K,t) = 1$, just as in [1]. Note that this set of equation, as opposed to the result in [1], does not explicitly depend on the semi-classical action $S(K,t)$. This was achieved by introducing a variable transformation $\pi \to \pi e^{iS(K,t)}$, which makes the equations easier to handle numerically because we do not have to do the integrations of eq. (1a) and (1b) in [1] at each time step. Also, the fact that now the evolution of the population n_m and coherence π do not contain the momentum gradient ∇_k [1] allows parallelization of the computation along the momentum axis. Concerning the dephasing time, we chose $T_2 = T_0/4$ in these calculations.

For the calculations in Fig. 3 (d) in the main text, we fitted the bands by parabolas as here only the interband contribution is calculated and higher orders of the conduction band as they are given in supplementary eq. (2) are not crucial, and the high-harmonic yield was calculated solving the integral given in eq. (4b) in [1] as follows: The integration in momentum space was replaced by a saddle-point approximation, integration over the ionization time was done numerically using a Gaussian quadrature routine, and the Fourier integral was performed as a fast Fourier Transform (FFT). From the many possible transitions between valence and conduction bands only the ones with dominant transition dipole was taken into account.

The intraband results in Fig. 3 (b) in the main text are based on fitting the conduction band by the expansion of higher order cosine terms as given in supplementary eq. (2) with the intraband current calculated as in [3].

Supplementary Note 3.

Prefactors needed in the calculation of the nonlinear susceptibilities using the experimental yield

The radiation yield emitted by a (non-relativistically) accelerated point charge is given by

$$Yield(t) = \frac{\big(q\cdot a(t)\big)^2}{6\pi\epsilon_0 c^3} \tag{5}$$

in the units of power. For the calculations with sapphire, we replace $q\cdot a(t)$ by the acceleration of the dipole $\frac{d^2}{dt^2}p(t)$ assuming that we have a constant polarization density over the focal volume V_{focal}. Keeping track of the coefficients that we get when performing the Fourier transform of the yield and when converting the field into intensity ($I = \frac{c\epsilon_0}{2}E_0^2$) we obtain

$$Yield_N = Yield(\omega = N\omega_0) = \frac{\left|V_{\text{focal}}\epsilon_0 a_N \chi^{(N)}(N\omega_0)^2\right|^2 \pi/22^N}{6\pi\epsilon_0 c^3 c^N \epsilon_0^N} I^N \quad (6)$$

Where SI units are used and where $a_N = 1/(2^{N-1})$. Here, the only unknown variable is $\chi^{(N)}$, which can be determined easily by comparing the prefactor of I^N in supplementary equation (6) with the prefactor A_N obtained in the perturbative fit.

WIEN2k and SBE calculations parameters

The struct file for the calculations of band structures using WIEN2K is given as follows.

```
Al2O3
R   LATTICE,NONEQUIV.ATOMS   2  167 R-3c
MODE OF CALC=RELA unit=bohr
  8.996041  8.996041 24.558125 90.000000 90.000000120.000000
ATOM  -1: X=0.35216000 Y=0.35216000 Z=0.35216000
          MULT= 4          ISPLIT= 4
      -1: X=0.64784000 Y=0.64784000 Z=0.64784000
      -1: X=0.85216000 Y=0.85216000 Z=0.85216000
      -1: X=0.14784000 Y=0.14784000 Z=0.14784000
Al         NPT=  781  R0=.000100000 RMT=   1.70000   Z:  13.00000
LOCAL ROT MATRIX:    1.0000000 0.0000000 0.0000000
                     0.0000000 1.0000000 0.0000000
                     0.0000000 0.0000000 1.0000000
ATOM  -2: X=0.94332000 Y=0.55668000 Z=0.25000000
          MULT= 6          ISPLIT= 8
      -2: X=0.05668000 Y=0.44332000 Z=0.75000000
      -2: X=0.55668000 Y=0.25000000 Z=0.94332000
      -2: X=0.44332000 Y=0.75000000 Z=0.05668000
      -2: X=0.25000000 Y=0.94332000 Z=0.55668000
      -2: X=0.75000000 Y=0.05668000 Z=0.44332000
O          NPT=  781  R0=.000100000 RMT=   1.70000   Z:   8.00000
```

LOCAL ROT MATRIX: 0.0000000 1.0000000 0.0000000

0.0000000 0.0000000 1.0000000

1.0000000 0.0000000 0.0000000

12   NUMBER OF SYMMETRY OPERATIONS

-1 0 0-0.00000000

0-1 0 0.00000000

0 0-1-0.00000000

1

0 0-1 0.00000000

-1 0 0-0.00000000

0-1 0-0.00000000

2

0-1 0-0.00000000

0 0-1-0.00000000

-1 0 0-0.00000000

3

0 1 0-0.00000000

0 0 1-0.00000000

1 0 0 0.00000000

4

0 0 1-0.00000000

1 0 0 0.00000000

0 1 0-0.00000000

5

1 0 0 0.00000000

0 1 0-0.00000000

0 0 1-0.00000000

6

0 0-1 0.50000000

0-1 0 0.50000000

-1 0 0 0.50000000

7

0 0 1 0.50000000

0 1 0 0.50000000

1 0 0 0.50000000

8

-1 0 0 0.50000000

0 0-1 0.50000000

0-1 0 0.50000000

9

0-1 0 0.50000000

-1 0 0 0.50000000

0 0-1 0.50000000

10

0 1 0 0.50000000

1 0 0 0.50000000

0 0 1 0.50000000

11

1 0 0 0.50000000

0 0 1 0.50000000

0 1 0 0.50000000

12

Initialization parameters of the calculation were set to the default settings of WIEN2K, which are summarized as follows.

RMT (Muffin-tin radius) reduction: no reduction

Exchange correlation potential: PBE functional

Energy separation between core/valence: -6.0 Ry

RKMAX (Matrix size given by RMT*Kmax): 7.0

Fermi energy calculation option: tetrahedron-method

Number of k points in full BZ: 1000

Additional calculations were done by specifying the k path along the Γ-A direction in order to obtain the band structure. The k path has been specified using xcrysden, a crystal visualisation software.

Supplementary references

1. Vampa, G. *et al*. Theoretical Analysis of High-Harmonic Generation in Solids. *Phys. Rev. Lett.* **113,** 073901 (2014).
2. Palik, E. D. *Handbook of Optical Constants of Solids*. (Academic, 1998).
3. Ghimire, S. *et al*. Observation of high-order harmonic generation in a bulk crystal. *Nat. Phys.* **7,** 138–141 (2011).
